# NADPH Oxidase-4 Overexpression Is Associated With Epithelial Ciliary Dysfunction in Neutrophilic Asthma

**DOI:** 10.1016/j.chest.2016.01.024

**Published:** 2016-02-02

**Authors:** Wing-Yan Heidi Wan, Fay Hollins, Louise Haste, Lucy Woodman, Robert A. Hirst, Sarah Bolton, Edith Gomez, Amanda Sutcliffe, Dhananjay Desai, Latifa Chachi, Vijay Mistry, Cédric Szyndralewiez, Andrew Wardlaw, Ruth Saunders, Christopher O’Callaghan, Peter W. Andrew, Christopher E. Brightling

**Affiliations:** a Institute for Lung Health, Department of Infection, Immunity & Inflammation, Glenfield Hospital, University of Leicester, Leicester; b Department of Infection, Immunity and Inflammation, University of Leicester, Leicester; c Centre for PCD Diagnosis and Research, Department of Infection, Immunity and Inflammation, RK Clinical Sciences Building, University of Leicester, Leicester; d Genkyotex, Geneva, Switzerland

**Keywords:** asthma, epithelial cells, NOX4, oxidative stress, ALI, air-liquid interface, CBF, ciliary beat frequency, DUOX, dual oxidase part of the NOX/DUOX family, GINA, Global Initiative for Asthma, mRNA, messenger RNA, NADPH, nicotinamide adenine dinucleotide phosphate, NOX, nicotinamide adenine dinucleotide phosphate oxidase part of the NOX/DUOX family, OVA, ovalbumin, ROS, reactive oxygen species

## Abstract

**Background:**

Bronchial epithelial ciliary dysfunction is an important feature of asthma. We sought to determine the role in asthma of neutrophilic inflammation and nicotinamide adenine dinucleotide phosphate (NADPH) oxidases in ciliary dysfunction.

**Methods:**

Bronchial epithelial ciliary function was assessed by using video microscopy in fresh ex vivo epithelial strips from patients with asthma stratified according to their sputum cell differentials and in culture specimens from healthy control subjects and patients with asthma. Bronchial epithelial oxidative damage was determined by 8-oxo-dG expression. Nicotinamide adenine dinucleotide phosphate oxidase (NOX)/dual oxidase (DUOX) expression was assessed in bronchial epithelial cells by using microarrays, with NOX4 and DUOX1/2 expression assessed in bronchial biopsy specimens. Ciliary dysfunction following NADPH oxidase inhibition, using GKT137831, was evaluated in fresh epithelial strips from patients with asthma and a murine model of ovalbumin sensitization and challenge.

**Results:**

Ciliary beat frequency was impaired in patients with asthma with sputum neutrophilia (n = 11) vs those without (n = 10) (5.8 [0.6] Hz vs 8.8 [0.5] Hz; *P* = .003) and was correlated with sputum neutrophil count (*r* = –0.70; *P* < .001). Primary bronchial epithelial cells expressed DUOX1/2 and NOX4. Levels of 8-oxo-dG and NOX4 were elevated in patients with neutrophilic vs nonneutrophilic asthma, DUOX1 was elevated in both, and DUOX2 was elevated in nonneutrophilic asthma in vivo. In primary epithelial cultures, ciliary dysfunction did not persist, although NOX4 expression and reactive oxygen species generation was increased from patients with neutrophilic asthma. GKT137831 both improved ciliary function in ex vivo epithelial strips (n = 13), relative to the intensity of neutrophilic inflammation, and abolished ciliary dysfunction in the murine asthma model with no reduction in inflammation.

**Conclusions:**

Ciliary dysfunction is increased in neutrophilic asthma associated with increased NOX4 expression and is attenuated by NADPH oxidase inhibition.

Normal mucociliary clearance is essential in pulmonary defense.^1^ Abnormal mucociliary clearance is a feature of asthma, particularly in severe disease, as a consequence of ciliary dysfunction.^2^ This ciliary dysfunction might contribute to the persistent inflammation and susceptibility to infection in the asthmatic airway, as evidenced by higher bacterial DNA level^3^, ^4^ and fungal colonization, in particular *Aspergillus fumigatus.*^5^

Importantly, asthma is a heterogeneous disease and in addition to the Th2-mediated eosinophilic paradigm, neutrophilic-predominant inflammation is a feature of one third of patients with asthma.^6^, ^7^, ^8^, ^9^ Although the cause of neutrophilic asthma is unclear, it is associated with increased presence of proinflammatory and Th1 cytokines in sputum and bacterial colonization.^10^ Pathogens can exert direct toxic effects on ciliary function or indirectly via oxidative stress,^11^ and stimuli such as infection,^12^ pollutants^13^ and proinflammatory mediators^14^ can induce production of reactive oxygen species (ROS). The nicotinamide adenine dinucleotide phosphate (NADPH) oxidase (NOX)/dual oxidase (DUOX) family plays an important role in the generation of ROS and contains seven members: NOX1-5 and DUOX1/2.^15^ DUOX expression in the bronchial epithelium has been reported,^16^, ^17^, ^18^ and DUOXs have been shown to be important for neutrophil recruitment to the airways.^19^ In addition, we have previously reported that NOX4 expression was increased in airway smooth muscle in asthma, leading to increased ROS production and intrinsic airway smooth muscle hypercontractility.^20^ Whether NOX/DUOX expression is altered in the bronchial epithelium and possibly contributes to an increased susceptibility to ciliary dysfunction in asthma is unknown.

We hypothesized that ciliary dysfunction in asthma is due to a combination of an intrinsic abnormality in ciliary function and airway inflammation. To test our hypothesis, we assessed: (1) the ciliary beat frequency (CBF) in fresh ex vivo epithelial cells from patients with asthma with and without sputum neutrophilia, (2) the role of NADPH oxidases in ciliary function and their specificity to the neutrophilic asthma phenotype, and (3) the effects of NADPH oxidase inhibition on ciliary function in a murine in vivo ovalbumin (OVA) sensitization and challenge model.

## Methods

A more detailed description of the methods is available in e-Appendix 1.

### Subjects

Patients with asthma and healthy control subjects were recruited from a single center (Glenfield Hospital). Asthma severity was defined by using the Global Initiative for Asthma (GINA) treatment steps (mild to moderate asthma, GINA steps 1-3; severe asthma, GINA steps 4-5).^21^ The study was approved by the Leicestershire Ethics Committee (UHL 10613), and patients gave their written informed consent.

### Epithelial Cells

Primary epithelial cells were isolated from bronchial brushes during bronchoscopy. Experiments were undertaken by using epithelial human bronchial epithelial cells, characterized by cytokeratin 5 and 14 (Abcam) expression.^22^ Ciliary function was assessed in ciliated air-liquid interface (ALI) cultures or fresh epithelial strips by using video microscopy as previously described.^23^, ^24^

### Immunohistochemistry and Immunofluorescence

Human bronchial biopsy specimens were embedded in glycomethacrylate.^20^ Sections were stained by using an 8-oxo-dG monoclonal antibody, anti-NADPH oxidase 4 antibody (Abcam), anti-DUOX1 (Abcam), and anti-DUOX2 (Millipore) or corresponding isotype control (DAKO and ImmunoStep). Staining intensity above isotype control for 8-oxo-dG expression was assessed by using a semiquantitative scoring ranging from none to low, moderate, or high staining (0-3). For NOX4 and DUOX1/2, staining intensity was measured in all areas of epithelium by thresholding using CellˆF software (Olympus). All assessments were made by an observer blinded to the subjects' clinical characteristics. Cytospins of human bronchial epithelial cells were labeled with polyclonal rabbit antibodies to NOX1 and NOX4 (4 μg/mL, Insight Biotechnology; 4 μg/mL, Abcam, respectively) or the corresponding isotype control (BD Bioscience). They were indirectly labeled with an R-Phycoerythrin-conjugated secondary antibody (AbD Serotec). Cells were counterstained with 4′,6′-diamidino-2 phenylindole (1 μg/mL; Sigma-Aldrich).

### RNA Extraction, Real-Time Quantitative Polymerase Chain Reaction, and Gene Array

Total RNA was extracted from epithelial ALI cultures by using an RNeasy Mini Kit with DNase I treatment (Qiagen). Complementary DNA synthesis and quantitative polymerase chain reaction were performed with a two-step real-time quantitative polymerase chain reaction kit (Invitrogen) by using the Chromo4 Real-Time Detector and Opticon Monitor 3 (Bio-Rad Laboratories). The internal normalizer gene was 18S RNA amplified with 18S primer forward (h18SRNA.891F:GTTGGTTTTCGGAACTGAGG) and 18S reverse primer (h18SRNA.1090R:GCATCGTTTATGGTCGGAAC); amplification of NOX4 was with primers forward (hNox4.598F:TGGCTGCCCATCTGGTGAATG) and reverse (hNox4.878R:CAGCAGCCCTCCTGAAACATGC). Primers (Operon MWG Biotech) and reaction mix (Invitrogen) were used as described previously.^20^ Relative quantification of NOX4 messenger RNA (mRNA) was performed by using the comparative 2^−ΔΔCt^ method and expressed as fold-change. The internal normalizer gene was 18S RNA.

RNA expression levels from basal epithelial cells were examined by using the Human Genome U133A probe array (GeneChip; Affymetrix). Hybridized biotinylated complementary RNA was stained with streptavidin phycoerythrin (Molecular Probes), scanned with a HP GeneArray Scanner (Hewlett-Packard). Image data from each microarray were individually scaled to an intensity of 200 by using GeneChip Operating Software (Affymetrix). Scaled average differences and absolute call data were exported to text files for further analysis.

### Intracellular ROS Assay

Intracellular ROS generation in human bronchial epithelial cells was quantified by using 5-(and-6)-carboxy-2′, 7′-dichlorofluorescein diacetate oxidation.^20^

### In Vivo Model of OVA Sensitization and Challenge

In a standard murine model of OVA sensitization and challenge protocol that was adapted from Caceres et al,^25^ airway inflammation, remodeling, and ciliary function were assessed. The University of Leicester Ethics Committee and the UK Home Office approved the experimental protocols.

### NOX1/4 Inhibitor GKT137831

To investigate the effects of NADPH oxidase inhibition in asthmatic epithelial cells and a murine model, we used GKT137831 (Genkyotex),^26^ a NADPH oxidase inhibitor that is selective for NOX1 and NOX4 vs NOX2, NOX3, and NOX5. GKT137831 has greater selectivity and potency for NOX4 than for NOX1. Its potential inhibitory effects on DUOX1/2 have not been fully explored.

### Statistical Analysis

Statistical analyses were performed by using PRISM version 6 (GraphPad Software). Parametric data were described as mean ± SEM; geometric mean ± 95% confidence intervals (CIs) were used for log normally distributed data, and nonparametric data are presented as median ± interquartile range. Comparisons between groups were assessed by using *t* tests (Mann-Whitney *U* test for nonparametric data). Comparisons across ≥ 3 groups were analyzed by using an analysis of variance (ANOVA) model (Kruskal-Wallis test for nonparametric data) with post hoc pairwise comparisons with the Tukey test (the Dunn test for nonparametric analyses). Sputum eosinophil (> 3%) and neutrophil (> 61%) counts were used as cutoffs to define inflammatory asthma phenotypes.^27^ Statistical analyses were performed as indicated in the figure legends, and *P* < .05 was taken as the level of statistical significance.

## Results

Clinical characteristics of the subjects who provided bronchial biopsy specimens for immunohistochemistry or epithelial brushings for ex vivo bronchial epithelial strips are shown Table 1.

**Figure 1:**
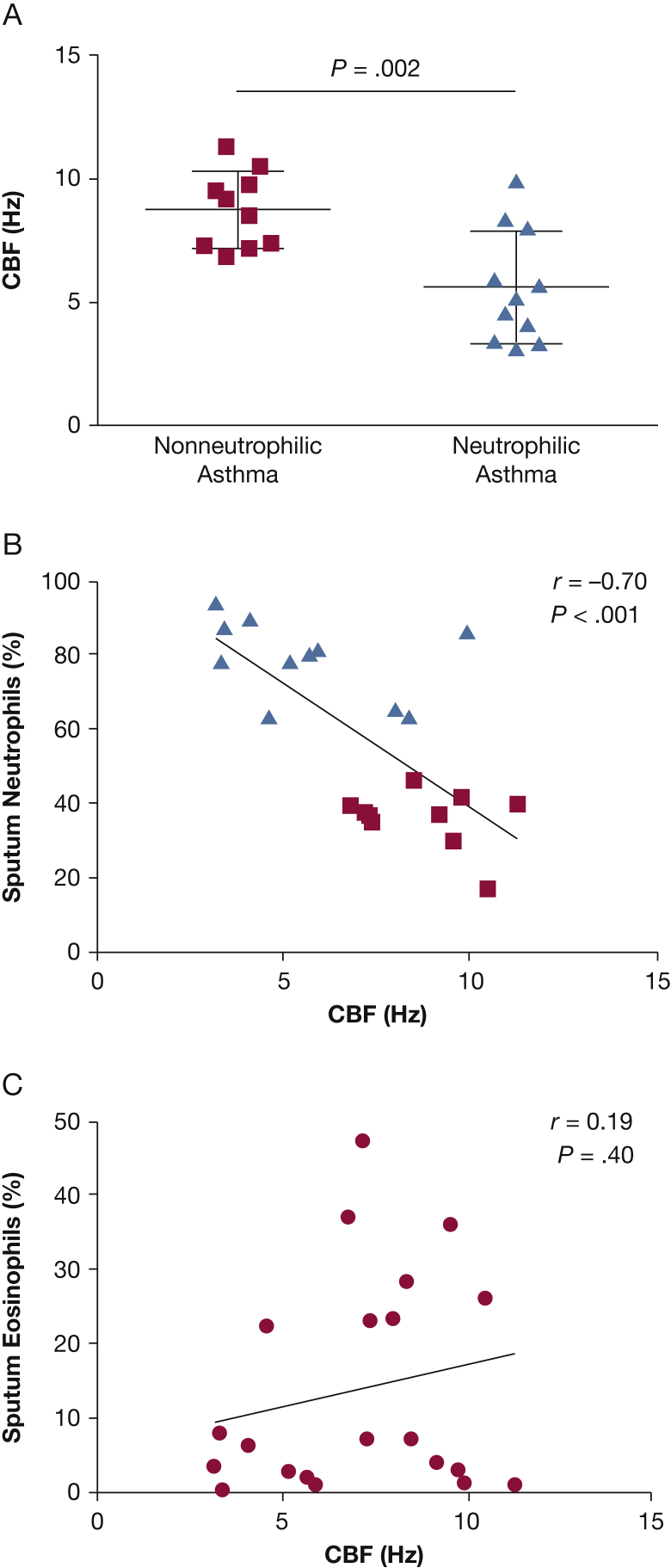
A-C, Baseline ciliary function of ciliated fresh ex vivo bronchial epithelial strips. (A) CBF was measured in fresh ex vivo epithelial strips from 10 patients with nonneutrophilic asthma and 11 patients with neutrophilic asthma. Comparisons were made by using unpaired *t* tests. (B) Correlation between CBF of the fresh ex vivo epithelial strips and sputum neutrophil count (n = 21). Patients with asthma with or without sputum neutrophilia (> 61%) are represented as triangles or squares, respectively. (C) The CBF of the fresh ex vivo epithelial strips and sputum eosinophil count is not correlated (n = 21). CBF = ciliary beat frequency.

CBF was reduced in patients with neutrophilic vs nonneutrophilic asthma (mean difference ± 95% CIs, –2.97 ± 0.87; *P* = .003) (Fig 1A) and was correlated with the percent sputum neutrophil count (*r* = –0.70; *P* < .001) (Fig 1B). Compared with our previously published CBF in healthy control subjects (10.7 ± 0.4 Hz),^2^ CBF was reduced in those patients with asthma with sputum neutrophilia (5.8 ± 0.6 Hz; *P* < .0001) but not in those without sputum neutrophilia (8.8 ± 0.5 Hz; *P* = .089). There was no relationship between sputum eosinophil count and CBF (*r* = 0.19; *P* = .40) (Fig 1C).

**Figure 2:**
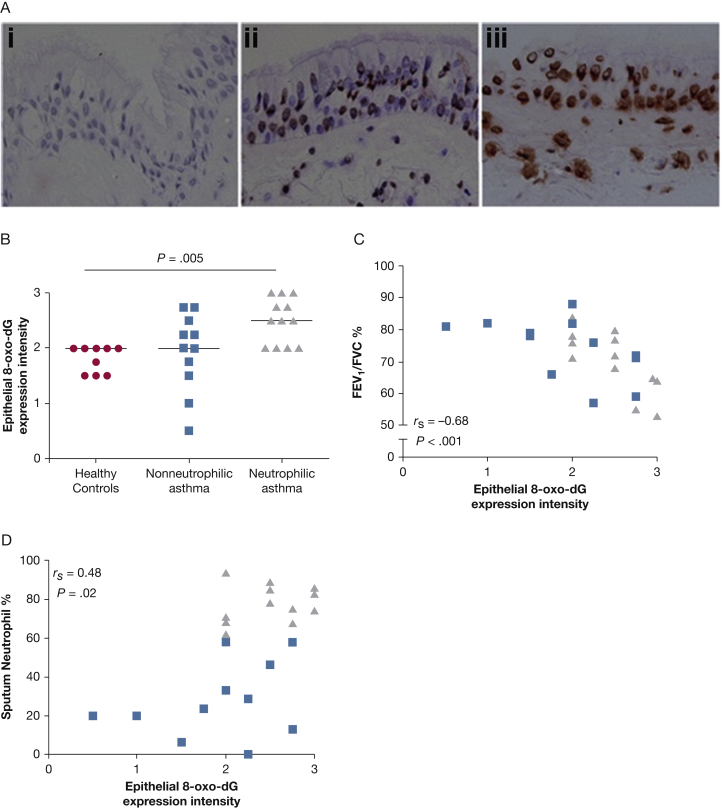
A-D, Oxidative damage (8-oxo-dG levels) in human bronchial epithelium in vivo. (A) Representative photomicrographs showing 8-oxo-dG staining in the epithelium in bronchial biopsy specimens from isotype control subjects (i); from a patient with asthma with positive staining of medium intensity (ii) and high intensity (iii) (×400). (B) Expression of 8-oxo-dG using a semiquantitative score (0 = none, 1 = low, 2 = moderate, 3 = high expression) in healthy control subjects (circles) and in patients with asthma with or without sputum neutrophilia (> 61%) (triangles or squares, respectively). Horizontal bars represent medians. Kruskal-Wallis test, *P* = .005. (C) and (D) Correlation of the expression of 8-oxo-dG with airflow obstruction and sputum neutrophil count, respectively (Spearman correlations are as shown).

Oxidative stress-induced DNA damage, represented by semiquantitative assessment of 8-oxo-dG expression, was increased in the bronchial epithelium in patients with neutrophilic asthma (Kruskal-Wallis test, *P* = .005) (Figs 2A, 2B) and in those with severe disease vs mild to moderate disease and healthy control subjects (Kruskal-Wallis test, *P* = .036; data not shown). The intensity of bronchial epithelial 8-oxo-dG expression was also correlated to airflow obstruction (*r*_s_ = –0.68; *P* < .001) (Fig 2C) and the percentage of sputum neutrophils (*r*_s_ = 0.48; *P* = .02) (Fig 2D).

Gene array analysis revealed the presence of NOX4 and DUOX1 and DUOX2 expression, but not the other NOX family members, in primary basal epithelial cells from ≥ 3 of the six donors with asthma. There was no significant difference in the proportion of healthy vs asthmatic donors who expressed NOX4, DUOX1, or DUOX 2, and their relative expression was not significantly different between cells from patients with asthma vs healthy control subjects (NOX4, 1.6-fold [0.79-3.1]; DUOX1, 0.87-fold [0.46-1.63]; and DUOX2, 1.12-fold [0.38-3.3]). The geometric mean (95% CI) percent β-actin expression was 0.07% (0.05-0.12) for NOX4, 0.69% (0.56-0.95) for DUOX1, and 0.45% (0.06-1.3) for DUOX2. We therefore focused our immunohistochemical analysis of bronchial biopsy specimens on NOX4 and DUOX1/2.

**Figure 3:**
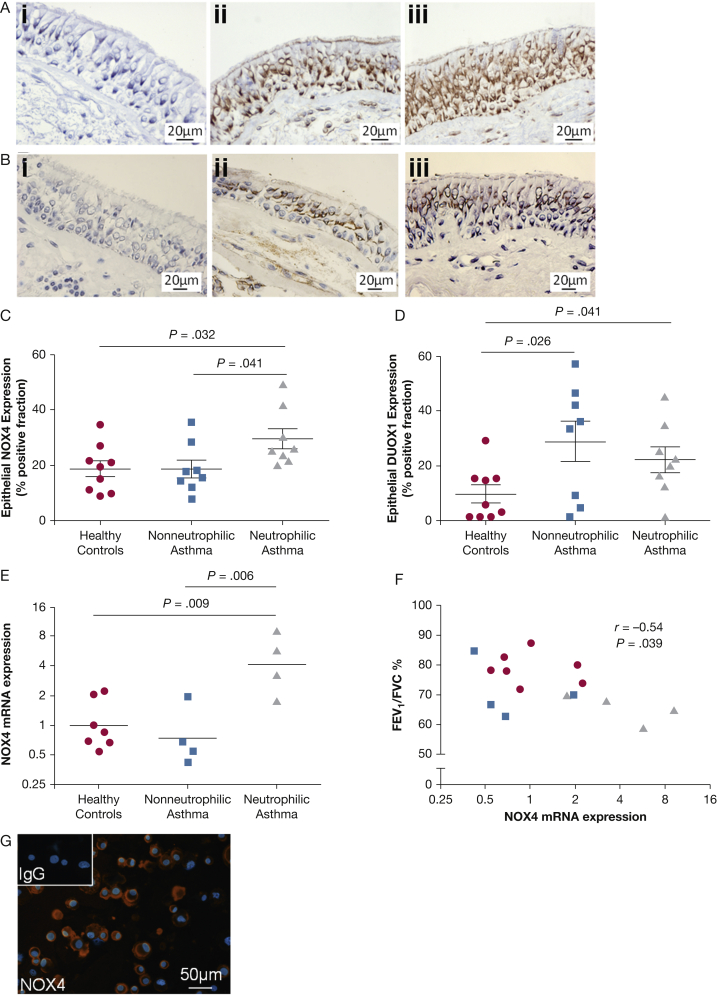
A-G, Bronchial epithelial nicotinamide adenine dinucleotide phosphate (NADPH) oxidase expression in asthma in vivo and in vitro. (A) Representative immunohistochemistry photomicrographs of (i) bronchial biopsy specimen stained with an isotype control and epithelial NOX4 protein expression in bronchial biopsy tissue from patients with (ii) nonneutrophilic and (iii) neutrophilic asthma. (B) Representative immunohistochemistry photomicrographs of (i) bronchial biopsy specimens stained with an isotype control and epithelial DUOX1 protein expression in bronchial biopsy tissue from patients with (ii) nonneutrophilic and (iii) neutrophilic asthma. Intensity of staining of (C) NOX4 and (D) DUOX1 was measured by using thresholding and expressed as percent area of positive staining. Bar represents mean (SEM). (E) NOX4 gene expression in air-liquid interface cultures, quantified by real-time quantitative polymerase chain reaction, in healthy control subjects (n = 7; circles), patients with nonneutrophilic asthma (n = 4; squares), and patients with neutrophilic asthma (n = 4; triangles). Analysis of variance and post hoc Tukey tests as shown. (F) Correlation between NOX4 gene expression in air-liquid interface cultures and airflow obstruction (Spearman correlation as shown). (G) Immunofluorescence staining of cytospun basal epithelial cells for NOX4. NOX4 is stained red, nuclei is stained blue, and the isotype control is shown as inset; ×20 magnification, scale bar represents 50 μm. mRNA = messenger RNA.

NOX4 protein expression in epithelial cells of tissue bronchial biopsy specimens was significantly increased in patients with neutrophilic asthma (29.5% ± 3.7%) compared with patients with nonneutrophilic asthma (18.5% ± 3.2%; *P* = .041) and with healthy control subjects (18.6% ± 2.9%; *P* = .032) (ANOVA, *P* = .040) (Figs 3A, 3C), and it was significantly correlated with sputum neutrophil count (*r* = 0.52; *P* =.042) but not with lung function. DUOX1 protein expression was increased in the epithelium in patients with nonneutrophilic (29.0% ± 7.4%) and neutrophilic (22.3% ± 4.7%) asthma vs healthy control subjects (9.8% ± 3.2%; *P* = .026 and *P* = .041, respectively); it was not differentially expressed in neutrophilic vs nonneutrophilic phenotypes (*P* = .465) (ANOVA, *P* = .048) (Figs 3B, 3D) and did not correlate with sputum neutrophil count (*r* = –0.06; *P* = .832). Although DUOX2 was expressed in a much lower percentage of epithelium, a significant increase was seen in patients with nonneutrophilic asthma (0.97% ± 0.29%; *P* = .026) but not neutrophilic asthma (0.58% ± 0.15%; *P* = .072) compared with healthy control subjects (0.27% ± 0.07%), and expression was not differentially expressed in neutrophilic vs nonneutrophilic phenotypes (*P* = .258) (ANOVA, *P* = .045) (e-Fig 1).

NOX4 mRNA in epithelial ALI cultures was significantly increased in neutrophilic asthma samples compared with healthy control samples (*P* = .009) and nonneutrophilic asthma samples (*P* = .0061) (ANOVA, *P* = .004) (Fig 3E). NOX4 mRNA expression, as with DNA damage, was correlated to airflow obstruction (*r* = –0.54; *P* = .039) (Fig 3F). In basal epithelial cells, NOX4 mRNA expression was similarly elevated, although not significantly, in the neutrophilic subtype compared with the health and the nonneutrophilic subtype (data not shown). At the protein level, basal epithelial cells expressed NOX4 by immunofluorescence (Fig 3G) but not NOX1 (data not shown).

**Figure 4:**
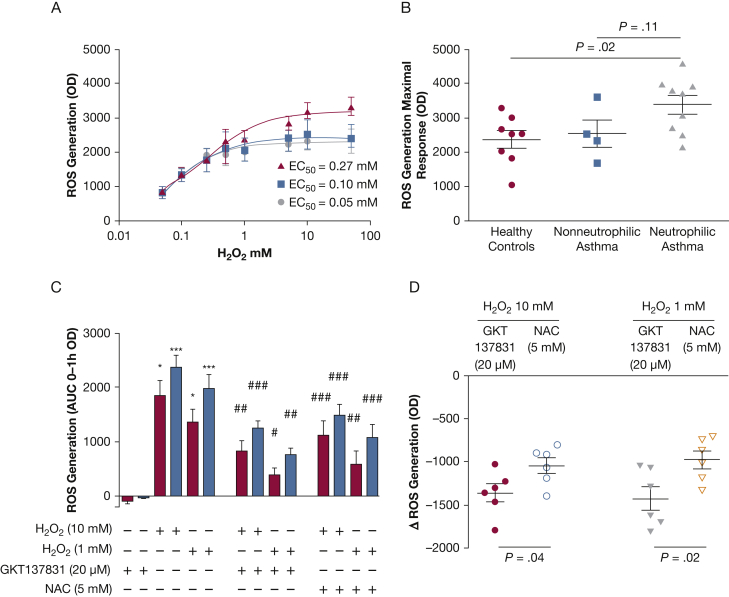
A-D, The role of NADPH oxidase in oxidant-induced reactive oxygen species (ROS) generation. (A) Generation of dose-dependent intracellular ROS, induced by H_2_O_2_, in human bronchial epithelial cells from healthy control subjects (n = 8; circles), patients with nonneutrophilic asthma (n = 4; squares), and patients with neutrophilic asthma (n = 9; triangles) quantified by using the 5-(and-6)-carboxy-2′, 7′-dichlorofluorescein diacetate assay. (B) Corresponding maximum dose response for individual subjects. (C) The effect of GKT137831 on H_2_O_2_-induced intracellular ROS generation in human bronchial epithelial cells from healthy control subjects (n = 3; open bars) and patients with asthma (n = 6; closed bars). *Comparison with corresponding GKT137831-only groups; # comparison with corresponding H_2_O_2_-only groups, *P* < .05; ## *P* < .01; ∗∗∗, ### *P* < .001. (D) Reduction of H_2_O_2_ induced-intracellular ROS generation in response to GKT137831 (20 μM) and NAC (5 mM). Unpaired *t* test, *P* < .05. EC_50_ = half maximal effective concentration; NAC = N-acetylcysteine; OD = optical density; ROS = reactive oxygen species.

Baseline ROS production did not differ between health and nonneutrophilic or neutrophilic asthma. However, ROS-induced ROS production in response to H_2_0_2_ stimulation increased in neutrophilic asthma compared with health and nonneutrophilic asthma as the concentration of H_2_0_2_ increased. Indeed, the maximal H_2_O_2_-induced intracellular ROS generation in basal epithelial cells was significantly increased in patients with neutrophilic asthma (mean ± SEM optical density, OD arbitrary units) (3,380 ± 270) compared with health (2,371 ± 252; *P* = .02) but not significantly increased compared with patients with nonneutrophilic asthma (2,542 ± 398; *P* = .11) (ANOVA, *P* = .037) (Figs 4A, 4B). The response to this maximum dose demonstrated a trend to a correlation in the percentage of sputum neutrophils (*r* = 0.4; *P* = .052). GKT137831 at 20 μM (Genkyotex) significantly reduced H_2_O_2_-induced (10 mM) intracellular ROS generation, in health from 1,849 ± 281 to 826 ± 194 (mean difference [95% CI], 1022 [–1407 to –638]; *P* = .001), and in asthma from 2,356 ± 246 to 1,247 ± 134 (–1,130 [–1,429 to –830]; *P* = .0002) (Fig 4C). GKT137831 similarly attenuated 1 mM H_2_O_2_-induced ROS generation. In patients with asthma, this reduction by GKT137831 (–1,362 ± 105) was greater than with N-acetylcysteine (5 mM) (–1,044 ± 90) (mean difference [95% CI], –318 [–626 to –10]; *P* = .04) (Fig 4D). The reduction in H_2_O_2_-induced (10 mM or 1 mM) ROS generation in response to GKT137831 or N-acetylcysteine was not significantly different between health and asthma.

**Figure 5:**
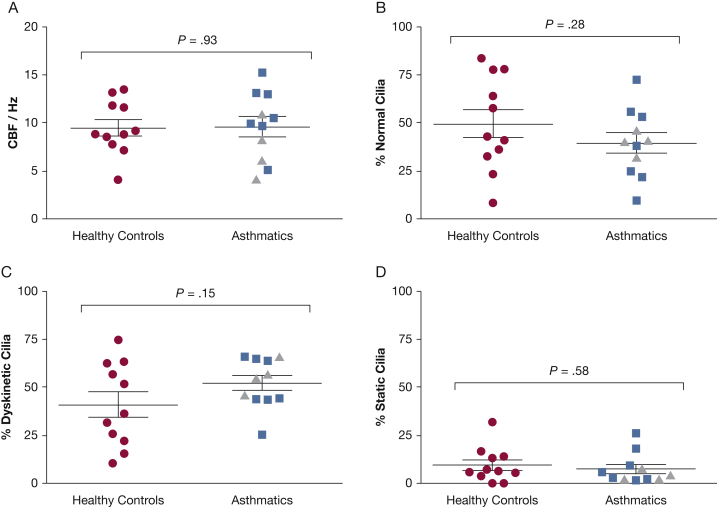
A-D, Ciliary function of human primary bronchial epithelial cell cultures. (A) CBF of ciliated epithelial cells from healthy control subjects (circles), patients with nonneutrophilic asthma (squares), and patients with neutrophilic asthma (triangles). Ciliary beat patterns were expressed in the percentage of (B) normally beating cilia, (C) dyskinetic cilia, and (D) static cilia. Unpaired *t* test, *P* values as shown. See Figure 1 legend for expansion of abbreviation.

CBF and pattern were assessed in primary ALI cultured epithelial cells from subjects with and without asthma by using high-speed video microscopy (Videos 1A-1D). In contrast to our ex vivo observations, there was no significant difference in the mean ± SEM CBF between health and disease according to the scraping method (difference between means, 0.12 ± 1.4 Hz; *P* = .93) (Fig 5A) or the overhead viewing method (difference between means, 0.80 ± 1.40; *P* = .57 [five healthy control subjects and 20 patients with asthma]; data not shown). There were no relationships with asthma severity (data not shown) or sputum neutrophil count (*r*_s_ = –0.32; *P* = .34). Beat patterns were similar in the healthy control and asthmatic groups, including percentage of normal cilia (50 ± 7% vs 40% ± 5%; *P* = .28) (Fig 5B), dyskinetic cilia (41% ± 7% vs 53% ± 4%; *P* = .15) (Fig 5C), and static cilia (9% ± 3% vs 7% ± 2%; *P* = .58) (Fig 5D). These findings, together with similar levels of ciliogenesis in culture between asthma and health (60% ± 6% vs 63% ± 4%; *P* = .72) and surface morphology index (1.6 ± 0.14 vs 1.7 ± 0.12; *P* = .54), suggest the bronchial epithelial cells from patients with asthma do not have a primary deficiency in differentiation.

**Figure 6:**
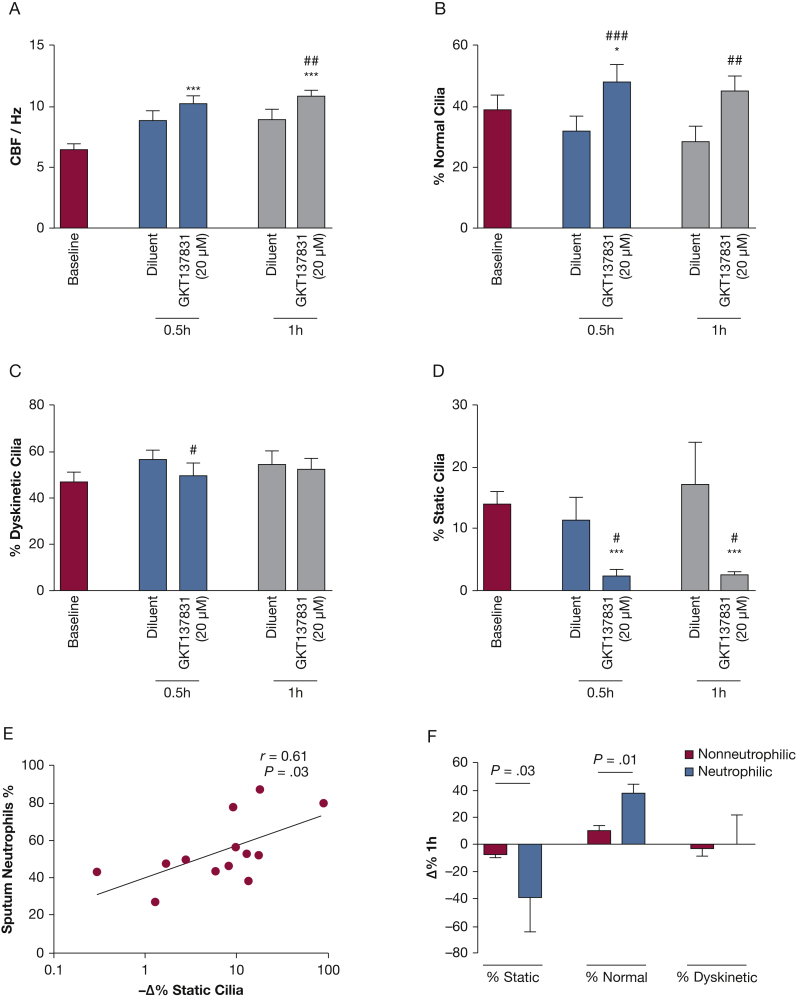
A-F, Effect of GKT137831 on ciliary function of fresh ex vivo bronchial epithelial strips. The effect of GKT137831 (20 μM) on the ciliary function over 1 h was assessed by using fresh asthmatic bronchial epithelial strips (n = 13) from bronchoscopy. Six to 10 side profiles were recorded per sample for analysis. Ciliary function was represented by CBF (A), and beat patterns are presented as percentage of (B) normal cilia, (C) dyskinetic cilia, and (D) static cilia at baseline (open bars), 0.5 h (light grey bars), and 1 h (dark grey bars). *Comparison with baseline; # comparison with corresponding diluent controls: paired *t* test, *P* < .05. (E) Correlation between percentage of sputum neutrophils and GKT137831 20 μM-induced absolute change as percentage of static cilia in 1 h. Pearson’s correlation, *P* < .05. (F) Improvement in GKT137831-induced beat patterns in nonneutrophilic and neutrophilic asthma subtypes. Unpaired *t* test, *P* values as shown. ## *P* < .01; ∗∗∗, ### *P* < .001. See Figure 1 legend for expansion of abbreviation.

To further establish the role of NADPH oxidase in ciliary dysfunction in asthma, we investigated the effect of GKT137831 on ciliary function of fresh bronchial epithelial strips studied within 1.5 h of bronchoscopy. These experiments were performed without any additional stimuli in contrast to the in vitro experiments described earlier. Using these fresh samples from patients with asthma, GKT137831 (5 μM and 20 μM) significantly increased CBF from baseline over the 1 h of incubation. GKT137831 20 μM at 1 h resulted in elevated CBF (10.9 ± 0.6 Hz), significantly different from baseline (6.4 ± 0.5 Hz) (mean difference [95% CI], 4.5 [3.5-5.5]; *P* < .0001) and from the diluent controls (8.9 ± 0.9 Hz, 2.0 [0.6-3.4]; *P* = .01) (Fig 6A). With GKT137831 20 μM, the percentage of normal cilia increased from 32% ± 5% to 48% ± 6% (mean difference [95% CI], 16% [10-22]; *P* < .0001) at 0.5 h and from 28% ± 5% to 45% ± 5% (mean difference [95% CI], 17% [6-27]; *P* = .01) at 1 h vs diluent (Fig 6B). The percentage of dyskinetic cilia was reduced from 57% ± 4% to 50 ± 5% (mean difference [95% CI], –7% [–14 to 0.3]; *P* = .04) at 0.5 h (Fig 6C) vs diluent. This improvement in CBF in response to NADPH oxidase inhibition was in fact due to cilia motility. NADPH oxidase inhibition lowered the proportion of static cilia compared with diluent, from 11% ± 4% to 2% ± 1% at 0.5 h (mean difference [95% CI], –9% [–16 to –2]; *P* = .01) and from 17% ± 7% to 2% ± 1% at 1 h (mean difference [95% CI], –15% [–29 to –0.6]; *P* = .04) (Fig 6D). Sputum neutrophil count was correlated to the GKT137831-induced reduction in the percentage of static cilia (*r* = 0.61; *P* = .03) (Fig 6E). It was also correlated to the percentage of normal cilia (*r* = 0.55; *P* = 0.05 [data not shown]). These improvements were significantly different in patients with neutrophilic vs nonneutrophilic asthma (Fig 6F).

**Figure 7:**
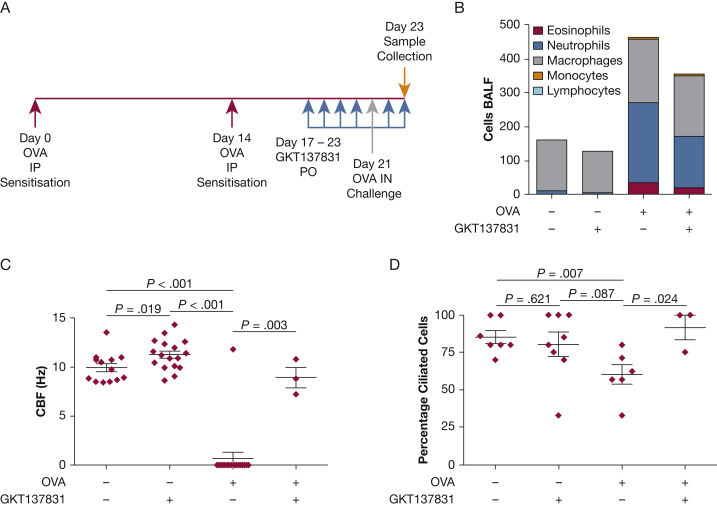
A-D, Effect of GKT137831 on ciliary function in an in vivo murine model of OVA sensitization and challenge. (A) Dosing regimen: up to 14 animals were used for each of the 4 conditions: (i) saline, (ii) GKT137831 (40 mg/kg) alone, (iii) model with saline control, or (iv) model in the presence of GKT137831 (40 mg/kg). (B) Differential cell counts of immune cells in the BALF of CBA/Ca mice were assessed at the time of culling. Counts are based on the number of immune cells counted in 10 fields of view at ×400 magnification. Lymphocytes were included in the counts but were too infrequent or absent to be included in color in the figure bars. Ciliary function is represented by (C) CBF and (D) percent ciliated cells. Analysis of variance and Kruskal-Wallis test for comparisons across groups with Tukey and Dunn tests for post hoc pairwise comparisons, respectively. BALF = bronchoalveolar lavage fluid; OVA = ovalbumin. See Figure 1 legend for expansion of other abbreviation.

In a murine model of OVA sensitization and challenge with the use of a standard protocol (Fig 7A), there was evidence of inflammation in the bronchoalveolar lavage, which was predominantly neutrophilic (Fig 7B) as well as ciliary dysfunction (Figs 7C, 7D). Administration of GKT137831 following OVA sensitization resulted in no significant change in bronchoalveolar lavage total inflammatory cell count (OVA sensitization alone vs GKT137831 alone, 303 [243-660] cells vs 240 [14-413] cells; *P* = .33) or cell differential. Following sensitization and challenge with OVA, the CBF was markedly impaired (*P* < .001 compared with baseline), and the addition of GKT137831 improved this finding almost back to baseline (*P* = .003). Similarly, the percentage of ciliated cells was reduced following sensitization and challenge with OVA compared with baseline (*P* = .007) and was restored following the addition of GKT137831 (*P* = .024).

## Discussion

To our knowledge, we report here for the first time that bronchial epithelial ciliary dysfunction in asthma is related to neutrophilic inflammation. This ciliary dysfunction, evident in vivo, does not persist in vitro, suggesting that the asthmatic airway environment is essential in the development and maintenance of ciliary dysfunction. In contrast to DUOX1, which was increased in asthma independent of inflammatory phenotype, and DUOX2, which was only increased in nonneutrophilic asthma, NOX4 expression was upregulated only in the bronchial epithelium from patients with asthma with neutrophilic inflammation in vivo. This increased NOX4 expression was also present in vitro with NADPH oxidase-dependent increased ROS generation in primary epithelial cells from patients with asthma with neutrophilic inflammation. Critically, NADPH oxidase inhibition completely abrogated ciliary dysfunction in ex vivo epithelial strips, as evidenced by restoration of CBF to the frequency we previously described in epithelial strips from healthy subjects^2^ and in an in vivo murine model of asthma with evidence of neutrophilic lung inflammation, but it did not significantly reduce this inflammation. Taken together, these findings support the view that ciliary dysfunction in asthma is both NADPH oxidase-dependent (in particular NOX4) and inflammation-dependent, with both necessary but neither sufficient, as persistent ciliary dysfunction was not observed in vitro despite increased NOX4 expression, and NADPH oxidase inhibition abolished ciliary dysfunction without affecting airway inflammation.

Noneosinophilic, and specifically neutrophilic, asthma is common^9^ but responds poorly to corticosteroid therapy.^7^ Neither the mechanisms nor the clinical management of neutrophilic asthma are fully understood. Oxidative stress and neutrophilia are often associated,^14^ and, consistent with this view, we found that neutrophilic asthma and airflow obstruction were associated with oxidative DNA damage, as evidenced by increased bronchial epithelial 8-oxo-dG expression assessed semiquantitatively. Critically, we extended our earlier observation that ciliary dysfunction is impaired in asthma and associated with increasing severity^2^ to demonstrate that this ciliary dysfunction is a particular feature of neutrophilic asthma. Normal ciliary function is a key component of innate immunity, and its impairment is likely to promote persistence of inflammation and increase risk of airway infection and colonization by pathogens.^3^, ^4^, ^10^ Indeed, macrolide antibiotics have demonstrated efficacy in noneosinophilic asthma,^28^ but concerns related to antibiotic resistance as a consequence of long-term widespread antibiotic use highlight the need for new therapies to treat neutrophilic asthma. The biologic relevance of the magnitude of CBF changes we observed in fresh bronchial strips following GKT137831 treatment must be addressed in future studies. However, following exposure to GKT137831, the CBF in the patients with asthma improved to a level we have reported previously in health^2^ and the improvement above diluent (2 Hz) was similar to the differences observed between health and COPD.^29^

NADPH oxidases play a critical role in ROS generation. NOX4 and DUOX1/2 expression in the epithelium was evident in asthma. DUOX1 was increased in asthma independent of inflammatory phenotype, and DUOX2 was only increased in nonneutrophilic asthma. In contrast, NOX4 was only increased in neutrophilic asthma. NOX4 plays a crucial role in regulating altered oxidative pathways in idiopathic pulmonary fibrosis^30^, ^31^, ^32^ and several nonrespiratory diseases,^26^, ^33^, ^34^ and its expression is upregulated by oxidative stress itself and inflammation. We found that increased ROS in the bronchial epithelium was a feature of neutrophilic asthma that persisted in vitro in association with increased NOX4 expression. Although NOX4 might be upregulated by neutrophilic inflammation, its overexpression can occur independently of the asthmatic environment. Importantly, ciliary dysfunction did not persist in vitro, suggesting this NOX4 upregulation was not itself sufficient to drive ciliary dysfunction but also required the presence of airway inflammation.

Beyond asthma, bronchial epithelial ciliary dysfunction is a fundamental abnormality in bronchiectasis,^35^ as well as in COPD.^36^ Antioxidant therapy, such as N-acetylcysteine, has been considered in these conditions; however, studies are inconsistent with benefits demonstrated in some studies^37^ but not in others.^38^, ^39^ The magnitude of benefit is also relatively small, possibly due to poor tolerance of high concentrations of the therapeutic agent, questionable bioavailability, and use of nonspecific antioxidant therapies that enhance the clearance of ROS rather than targeting ROS generation. Our findings suggest that NAPDH oxidase inhibition, which reduces intracellular ROS generation, improves ciliary dysfunction and thus extends the potential role of NADPH oxidases (and particularly NOX4) in asthma beyond the described impact of upregulated NOX4 on airway smooth muscle hypercontractility.^20^

A major limitation of our study is that although GKT137831 has good specificity and potency for NOX4, it also has effects on NOX1 and is likely to exert effects on DUOX, as has been observed with similar inhibitors in this class (eg, GKT136901).^40^ Our findings support a key role for NOX4; the expression of this NOX family member was increased in neutrophilic asthma and associated with ciliary dysfunction. However, we cannot fully exclude possible contributions from other mechanisms, including other members of the NOX family (particularly DUOX1/2). In addition, due to the considerable homology in the amino acid sequence of DUOX1 and DUOX2, the DUOX2 antibody may cross-react with DUOX1. However the amount of staining seen with the DUOX2 antibody is considerably less than that with the DUOX1 antibody. Thus, we are confident that DUOX2 is not highly expressed in the bronchial epithelium. One further potential limitation of our study is the lack of direct in vivo human data demonstrating that NADPH oxidase inhibition modifies ciliary function and inflammation. However, we have confirmed the importance of NADPH oxidase in ciliary dysfunction in fresh ex vivo primary cells studied immediately after obtaining the cells at bronchoscopy, and we supported the observations by using an in vivo murine model of asthma with neutrophilic lung inflammation. This asthma model clearly showed that NADPH oxidase inhibition markedly improved ciliary function but did not significantly attenuate lung inflammation. These findings suggest that although the presence of inflammation might be important for NOX4 activation, it is the consequent ROS generation rather than the inflammation per se that is critical in ciliary dysfunction.

## Conclusions

Bronchial epithelial cells from patients with asthma with neutrophilic airway inflammation exhibit heightened NOX4 expression and ROS generation. NADPH oxidase inhibition in an in vivo murine model of asthma improved ciliary function, which was also confirmed in ex vivo bronchial epithelial cells from patients with neutrophilic asthma. These findings support the view that NADPH oxidase inhibition (and in particular NOX4) might present a new stratified approach for the treatment of neutrophilic asthma.

## Figures and Tables

**Table 1:** Clinical Characteristics of Subjects Who Provided Bronchial Epithelial Cells for Ex Vivo Studies or Bronchial Biopsy Sampling

Characteristic	Ex Vivo Epithelial Cell Donors			Bronchial Biopsy Sample Donors		
Healthy Control (n = 8)	Neutrophilic (n = 11)	Nonneutrophilic (n = 10)	Healthy Control (n = 17)	Neutrophilic Asthma (n = 20)	Nonneutrophilic Asthma (n = 20)
Sex (male/female)	5/3	7/4	7/3	12/5	12/8	5/15
Age, y	52 ± 6	58 ± 4	57 ± 3	46 ± 4	55 ± 2	49 ± 3
Current/ex-/never smoker	0/4/4	0/4/7	0/2/10	0/1/16	0/2/18	0/0/20
Pack-years^a^	8 ± 5	3 ± 2	1 ± 1	0 (0-1)	0 (0-4)	0 (0-0)
Predicted FEV_1_, %	96 ± 4	80 ± 7	82 ± 6	96 ± 4	82 ± 5	79 ± 6
FEV_1_/FVC, %	78 ± 2	59 ± 5	62 ± 5	78 ± 2	67 ± 3	69 ± 3
Blood total IgE, kU/mL^a^	NA	139 (68-438)	172 (122-665)	NA	204 (36-1318)	243 (150-653)
Blood eosinophils, kU/mL^a^	NA	0.22 (0.14-0.56)	0.28 (0.19-0.40)	NA	0.47 (0.17-0.55)	0.23 (0.17-0.58)
Treatment (μg/24 BDP)^a^^,^^b^	NA	1,000 (800-2,000)	1,600 (750-2,000)	NA	1,600 (1,200-2,000)	1,600 (1,600-2,000)
GINA treatment step (1-3 or 4/5)	NA	4, 7	1, 9	NA	5, 15	8, 12
No. with atopy	NA	5	7	NA	15	16
Age of disease onset, y	NA	38 ± 7	26 ± 5	NA	22 ± 8	25 ± 6
Sputum characterization						
Eosinophils, %^a^	NA	3.5 (1.3-22.5)	15.2 (3.8-36.5)	NA	2.0 (0.3-5.2)	9.0 (0.4-23.7)
Neutrophils, %^a^	NA	80.0 (65.0-87.2)	37.2 (3.8-36.5)	NA	76.7 (67.9-85.8)	35.8 (20.0-46.5)

Data are presented as mean ± SD unless otherwise indicated. GINA = Global Initiative for Asthma; IgE = immunoglobulin E; NA = not applicable.

a Median (interquartile range).

b Beclomethasone dipropionate (BDP) equivalence.

## Supplementary Data

e-Online Data

Video 1A

Video 1B

Video 1C

Video 1D
