# Supplementary material for: NADPH Oxidase-4 Overexpression Is Associated With Epithelial Ciliary Dysfunction in Neutrophilic Asthma
Source: Chest. 2016 Feb 2;149(6):1445–59. doi: 10.1016/j.chest.2016.01.024 (PMC4893823; doi:10.1016/j.chest.2016.01.024)
Supplement: e-Online Data [file mmc1.pdf]

CHEST Online Supplement

NADPH Oxidase-4 Overexpression Is Associated With Epithelial Ciliary Dysfunction in Neutrophilic Asthma

Wing-Yan Heidi Wan, PhD; Fay Hollins, PhD; Louise Haste, PhD; Lucy Woodman, PhD; Robert A. Hirst, PhD; Sarah Bolton, PhD; Edith Gomez, PhD; Amanda Sutcliffe, PhD; Dhananjay Desai, PhD; Latifa Chachi, PhD; Vijay Mistry, BSc; Cédric Szyndralewiez, PhD; Andrew Wardlaw, PhD; Ruth Saunders, PhD; Christopher O'Callaghan, PhD; Peter W. Andrew, PhD; and Christopher E. Brightling, PhD, FCCP

CHEST 2016; 149(6):1445-1459

Online supplements are not copyedited prior to posting and the author(s) take full responsibility for the accuracy of all data.

© 2016 AMERICAN COLLEGE OF CHEST PHYSICIANS. Reproduction of this article is prohibited without written permission from the American College of Chest Physicians. See online for more details. **DOI:** 10.1016/j.chest.2016.01.024

e-Appendix 1.

METHODS

Subjects

Asthmatic subjects and healthy controls were recruited from a single centre Glenfield Hospital, Leicester, UK. Asthma severity was defined by the Global Initiative for Asthma (GINA) treatment steps (mild-moderate GINA 1-3, severe GINA 4-5)[E1]. Clinical details, including demographics, smoking status, treatment, atopic status defined as one or more positive skin prick test (wheal >2mm above negative control) to common aeroallergens (cat, dog, grass, trees, *Dermatophagoides pteronyssinus* and *Aspergillus*), spirometry, methacholine challenge, blood total IgE and eosinophil levels, and sputum differential cell counts were recorded. Healthy controls had no history of asthma and normal lung function. The study was approved by the Leicestershire Ethics Committee and patients gave their written informed consent.

Cells

Primary epithelial cells were isolated from bronchial brushes during bronchoscopy. Cells were plated onto 1% (v/v) PureCol (Inamed Biomaterials, Nutacon, The Netherlands) -coated surface for basal cells to expand to confluence as submerged cultures in bronchial epithelial growth medium (BEGM) (Lonza Verviers, Belgium) supplemented with 0.3% (w/v) Fungizone®antimycotic (Gibco, Invitrogen, Paisley, UK) and 1% (v/v) antibiotic-antimycotic (AA) (Gibco). Basal cells were then raised to air-liquid interface (ALI) on Transwell® inserts (Corning, Lowell, MA) using ALI medium (50:50 BEGM:DMEM (Gibco), supplemented with 0.3% (w/v) Fungizone® Antimycotic (250µg/mL amphotericin B) (Gibco), 1% (v/v) Antibiotic/Antimycotic (10,000 units/mL of Penicillin, 10,000µg/mL of Streptomycin and 25µg/mL of Amphotericin B) (Gibco) and 100nM retinoic acid (Sigma)). Ciliated cultures with high ciliogenesis (proximately 2 weeks after the first observable cilia) were used for experiments. Experiments were undertaken using epithelial basal cells, ciliated ALI cultures, or fresh epithelial brushes.

Epithelial cell characterization

Basal epithelial cells were characterized to determine expression of cytokeratins 5 and 14 (Abcam)[E2]. Epithelial basal cells were cultured in 1% (v/v) PureCol-coated-8-wells permanox chamber slides (Lab-Tek, NalgeNunc) to confluence. Cells were fixed with ice-cold methanol (200µl/well) for 20 min on ice, followed

Online supplements are not copyedited prior to posting and the author(s) take full responsibility for the accuracy of all data.

by 10 mins air-drying. Cells were blocked in 3% (w/v) BSA-PBS for 1h. Cell cytoplasm was then stained with 1.6μg/ml human anti-cytokeratins (CKs) 5 and 14 antibody [LH8] (Abcam, Cambridge, UK) or isotype control (Dako, Ely, UK) (1.6μg/ml) for 1.5h, followed by secondary FITC-antibody (Dako) probing for 1.5h in the dark. Nuclei were stained with DAPI (Sigma) for 10s. 1% (w/v) BSA-PBS was used as the diluent. Three washes were done using 0.05% (v/v) Tween 20-PBS between each step. Images were obtained by x10 immunofluorescence microscopy and analysed by software OpenLab (PerkinElmer, Massachusetts).

Epithelial ciliary function

Ciliary function was assessed as previously described[E3]. CBF and beat patterns were enumerated from an overhead, or side view following scraping strips of ciliated cells from ALI cultures, or in fresh cell suspension (Video S1). For ALI cultures, strips of ciliated cells devoid of mucus were removed from the membrane insert and resuspended in 1ml M199 medium (Gibco). 100μl cell suspension was added to a chamber slide and placed on a heated platform (37ºC) of a microscope fitted with high-speed digital camera (DEL Imaging Systems, Cheshire, CT), linked to the software Motion Studio. Using x1000 oil immersion microscopy, videos of the side profiles of ciliated surfaces were recorded (500 frames/second for 2 s). 6 to 10 side profiles were recorded per sample. Videos could be played back, frame by frame, from which CBF (Hz) was enumerated. Beat patterns were categorised as normally beating (smooth beating with bendable tips), dyskinetic (stiff, disturbed beating with tip fails to bend along the ciliary shaft) and static. The level of ciliogenesis, and epithelial surface morphology (index of 1 (a smooth surface), 2 (relatively smooth surface with little cell protrusion), or 3 (irregular cell surface with cell protrusion)), were also assessed. Assessments were made by a single, blinded observer.

Immunohistochemistry and immunofluorescence

Human airway biopsies were obtained by bronchoscopy and embedded in glycomethacrylate (GMA)[E4]. Sequential 2 μm sections were cut and stained using an 8-oxo-dG monoclonal antibody (chromogen: AEC, mouse monoclonal, 20 μg/ml; Abcam), or isotype control (DAKO) and levels in airway epithelium assessed using a semi-quantitative scoring from none to high staining (0-3). Assessors were blinded to clinical characteristics. 2 μm sections were also stained with anti-NADPH oxidase 4 antibody (chromogen: DAB; rabbit monoclonal, 11μg/ml; Abcam), anti-DUOX1 antibody (chromogen: DAB; goat polyclonal, 1μg/ml; Abcam) and anti-DUOX2 antibody (chromogen: DAB; mouse monoclonal, 10μg/ml; Millipore, Watford, UK) or corresponding isotype controls (Immunostep or Dako). For mouse antibodies the EnVision FLEX kit

Online supplements are not copyedited prior to posting and the author(s) take full responsibility for the accuracy of all data.

(Dako) was used. Staining intensity above isotype control was measured in all areas of epithelium using thresholding using Cell^F software (Olympus, UK), where the thresholding levels set on Hue, Saturation and Intensity scales by an observer blinded to the subjects' clinical characteristics. Cytospins of HBEC were prepared and labelled with polyclonal rabbit antibodies to NOX1, NOX4 (4μg/mL; Insight Biotechnology, Wembley, UK, 4μg/mL; Abcam, Cambridge, UK, respectively) or corresponding isotype control (BD Bioscience, Oxford, UK) and indirectly labelled with an R-phycoerythrin conjugated secondary antibody (AbD Serotec, Kidlington, UK). Cells were counterstained with 4′,6′-diamidino-2 phenylindole (DAPI) (1μg/mL; Sigma, Gillingham, Dorset, UK). Images were taken on a Zeiss Axio Imager Z2 microscope with Zen software (Zeiss, Cambridge, UK).

RNA extraction, RT-qPCR and gene array

Total RNA was extracted from epithelial ALI cultures using a RNeasy Mini Kit with DNaseI treatment (Qiagen). cDNA synthesis and qPCR were done with a two-step RT-qPCR kit (Invitrogen) using a Chromo4 Real-Time Detector and Opticon Monitor 3 (BioRad). The internal normaliser gene was 18S RNA amplified with 18s primer forward (h18SRNA.891F:GTTGGTTTTCGGAACTGAGG) and 18s reverse primer (h18SRNA.1090R:GCATCGTTTATGGTCGGAAC); amplification of NOX4 was with primers forward (hNox4.598F:TGGCTGCCCATCTGGTGAATG) and reverse (hNox4.878R:CAGCAGCCCTCCTGAAACATGC). Primers (Operon MWG Biotech, Milton Keynes, U.K.) and reaction mix (Invitrogen) were used as described previously[E4]. Efficiency curves were determined for 20ng and 1ng cDNA as the input material for the NADPH oxidase (NOX) 4 gene and 18S gene, as the housekeeping gene, respectively in ALI cultures. Negative controls showed no gene expression. Relative quantification of Nox4 mRNA was done using the comparative $2^{-\Delta\Delta Ct}$ method and expressed as fold change. The internal normalizer gene was 18S RNA.

RNA expression levels from basal epithelial cells were examined using the Human Genome U133A probe array (GeneChip, Affymetrix, Santa Clara, CA, USA). Hybridized biotinylated cRNA was stained with streptavidin-phycoerythrin (Molecular Probes, Eugene, OR, USA), scanned with a HP Gene Array Scanner. All Affymetrix U133 plus 2.0 GeneChip® arrays passed the quality control characteristics. Image data from each microarray were individually scaled to an intensity of 200 using GCOS (Affymetrix). Scaled average difference and absolute call data were exported to text files for further analysis.

Online supplements are not copyedited prior to posting and the author(s) take full responsibility for the accuracy of all data.

Intracellular ROS assay

Intracellular ROS generation in basal cells was quantified using 5-(and-6)-carboxy-2′, 7′-dichlorofluorescein diacetate (DCF-DA) oxidation[E4]. Epithelial basal cells were incubated with DCF-DA stain at 37°C for 30 min, followed by stimulation with hydrogen peroxide (ranged 50mM to 50μM) for up to 1 h. PBS was used as the diluent. Cells stained with DCF-DA only were used as the control. Fluorescence was measured at excitation wavelength 485nm; emission 520nm, gain at 1100, using a NOVOStar microplate reader (BMG Labtech, Aylesbury, UK).

Effect of NOX1/4 inhibitor GKT137831

GKT137831 (Genkyotex, Switzerland) is a NADPH oxidase inhibitor which is selective for NOX1 and 4 versus NOX2, 3, and 5. GKT137831 has a 1.7 fold greater affinity and 1.5 fold greater potency for NOX4 than NOX1 [E5]. Its potential inhibitory effects upon DUOX1/2 have not been fully explored.

GKT137831 was used to investigate the role of NADPH oxidases in asthmatic epithelial cells. To investigate its effect on intracellular ROS generation, basal cells were incubated with GKT137831 only (20μM), H_2O_2 (10mM and 1mM) only, or in combination, at 37°C for 30 min or 1 hour prior to fluorescence measurements. NAC (5mM) was used as the positive control; corresponding diluents were used as the negative controls. To determine its effect on ciliary function, fresh asthmatic epithelial cells from bronchoscopy were incubated with GKT137831 (5μM and 20μM) at 37°C for 30 min or 1 hour prior to video-microscopy.

In vivo model of ovalbumin sensitization and challenge

In a standard murine model of ovalbumin (OVA) sensitisation and challenge protocol[E6], airway inflammation, and ciliary function were assessed. The University of Leicester Ethical Committee and the U.K. Home Office approved the experimental protocols.

For this combined model of pneumococcal infection and OVA sensitisation and challenge female outbred CBA/CaOlaHsd (CBA/Ca) mice were obtained from Harlan Olac (Bicester, UK). Mice were used when they were at least 9 weeks old. Before use, mice were kept for at least one week, under standard conditions in

Online supplements are not copyedited prior to posting and the author(s) take full responsibility for the accuracy of all data.

the University of Leicester's Division of Biomedical Services, with access to water and food *ad libitum.* All studies were performed in accordance with a U.K. Home Office licence (60/4327) and were approved by the University of Leicester Ethics Committee. Animals were assessed for visible signs of disease[E6] and were culled at pre-determined time points or if mice became severely lethargic.

On days 0 and 14 CBA/Ca mice were sensitised to OVA (100µg, intraperitoneal injection, Sigma, UK) adsorbed onto 1mg aluminium hydroxide (Alum, Fisher, UK) in physiological saline (Baxter, UK) in a volume of 300µl. On day 17, mice were lightly anaesthetised with a mixture of oxygen and 2.5% (v/v) Isofluorane (Abbott Laboratories, Maidenhead, UK) and $4x10^6$ CFU *S. pneumoniae* strain LgSt215 suspended in 30µl phosphate buffered saline (PBS) (Oxoid, UK) was instilled in droplets across both nares. On days 24 to 30, mice were given by oral gavage 40mg/kg GKT137831 in 1.2% (w/v) methylcellulose (Sigma, UK,) and 0.1% (w/v) polysorbate 80 (Sigma, UK) in distilled water). On day 28, mice were challenged intranasally with OVA (1mg/50µl physiological saline) and culled on day 30 with an intraperitoneal injection of 250µl Pentoject (20% (v/v) sodium pentobarbital (Pharmasol Ltd, Andover, UK). Death was confirmed by non-responsiveness to noxious stimuli (hind paw pinch) and exsanguination. Viable counts and differential cell counts were determined in the bronchoalveolar lavage fluid (BALF) (3 x 300µl) For viable counts BALF was plated onto blood agar supplemented with 5% (v/v) defibrinated Horse blood (Oxoid, UK) and incubated overnight at 37 °C.

For differential cell counts, BALF was centrifuged at 1850*g* for 10 minutes, the pellet was re-suspended in 200µl PBS and 50µl of suspension centrifuged at 160*g* for 3 minutes in a Cytospin Slide centrifuge (Shandon Southern Products Ltd, UK). Slides were allowed to dry overnight and then stained using a REASTAIN Quick-Diff kit (Reagena, Finland), according to the manufacturer's instructions. Cells in 10 fields-of-view were counted.

Ciliary beat frequency was assessed in bronchial rings using video-microscopy as described above for human epithelial cell strips. Bronchial rings were harvested from tracheas that had been embedded in 4% (v/v) UltraPure low melting point agarose (Sigma, UK), tracheas were then cut into 300µm sections on a temperature-controlled vibrotome (Campden Instruments, Leicester, UK). Sectioned rings were placed into Gibco Medium 199, pH 7.3 (Invitrogen, Paisley, UK) containing an antibiotic solution (streptomycin 50µg/ml, penicillin 50µg/ml, Gibco, UK) until used as described above.

Online supplements are not copyedited prior to posting and the author(s) take full responsibility for the accuracy of all data.

Statistical analysis

Statistical analyses were performed using PRISM version 6 (San Diego, CA). Parametric data were described as mean±SEM, geometric mean ±95% confidence interval for log normally distributed data and non-parametric data as median±interquartile range. Comparisons between groups were assessed by T-test (Mann-Whitney for non-parametric data). Comparisons across 3 or more groups were analysed by ANOVA (Kruskal-Wallis test for non-parametric data) with post hoc pairwise comparisons by Tukey's (Dunn's for non-parametric analyses). Sputum eosinophil (>3%) and neutrophil (>61%) were used as cut-offs to define inflammatory asthma phenotypes.[27] Statistical analyses were performed as indicated in the figure legends and $P<0.05$ was taken as the level of statistical significance.

Online supplements are not copyedited prior to posting and the author(s) take full responsibility for the accuracy of all data.

REFERENCES

E1. Global Strategy for Asthma Management and Prevention. Global Initiative for Asthma (GINA) 2014. http://www.ginasthma.org. Updated April 2015. Accessed 1st October 2015.

E2. Hicks W, Hall L, Sigurdson L, et al. Isolation and characterization of basal cells from human upper respiratory epithelium. *Exp Cell Res*. 1997;237:357-63.

E3. Chilvers MA, O'Callaghan C. Analysis of ciliary beat pattern and beat frequency using digital high speed imaging: comparison with the photomultiplier and photodiode methods. *Thorax*. 2000;55:314-7.

E4. Sutcliffe A, Hollins F, Gomez E, et al. Increased nicotinamide adenine dinucleotide phosphate oxidase 4 expression mediates intrinsic airway smooth muscle hypercontractility in asthma. *Am J Respir Crit Care Med*. 2012;185:267-74.

E5. Aoyama T, Paik YH, Watanabe S, et al. Nicotinamide adenine dinucleotide phosphate oxidase (nox) in experimental liver fibrosis: GKT137831 as a novel potential therapeutic agent. *Hepatology*. 2012;56:2316-27.

E6. Mitchell TJ, Paterson GK. Mouse models of Pneumococcal infection. In: Hakenbeck R, Chhatwal S, editors. Molecular Biology of Streptococci. Horizon Bioscience, Oxford Univ. Press; 2007. p. 543.

Online supplements are not copyedited prior to posting and the author(s) take full responsibility for the accuracy of all data.

FIGURE LEGENDS

e-Figure 1. DUOX2 staining in bronchial biopsies.

(A) Representative immunohistochemistry photomicrographs of (i) bronchial biopsy stained with isotype control, and (ii) epithelial DUOX2 protein expression in a bronchial biopsy from an asthmatic subject. (B) Intensity of staining of DUOX2 was measured in biopsies from healthy controls, non-neutrophilic asthmatics and neutrophilic asthmatics using thresholding and expressed as percentage area of positive staining. Bar represents mean (SEM).

Video 1. Example videos of ciliary function analysis.

Videos show the side profiles of ciliated human airway epithelial cell cultures at a non-stimulated status. Videos were recorded at a speed of 500 fps using videomicroscopy (100x oil immersion microscopy with a high-speed digital camera (DEL Imaging Systems, Cheshire, CT), linked to the software Motion Studio), and played back in slow motion. (A) Normal beating cilia with morphology index 1. (B) Normal beating cilia with morphology index 2. (C) Dyskinetic cilia with morphology index 3. (D) Static cilia with morphology index 2.

Online supplements are not copyedited prior to posting and the author(s) take full responsibility for the accuracy of all data.

CHEST Online Supplement

e-Figure 1

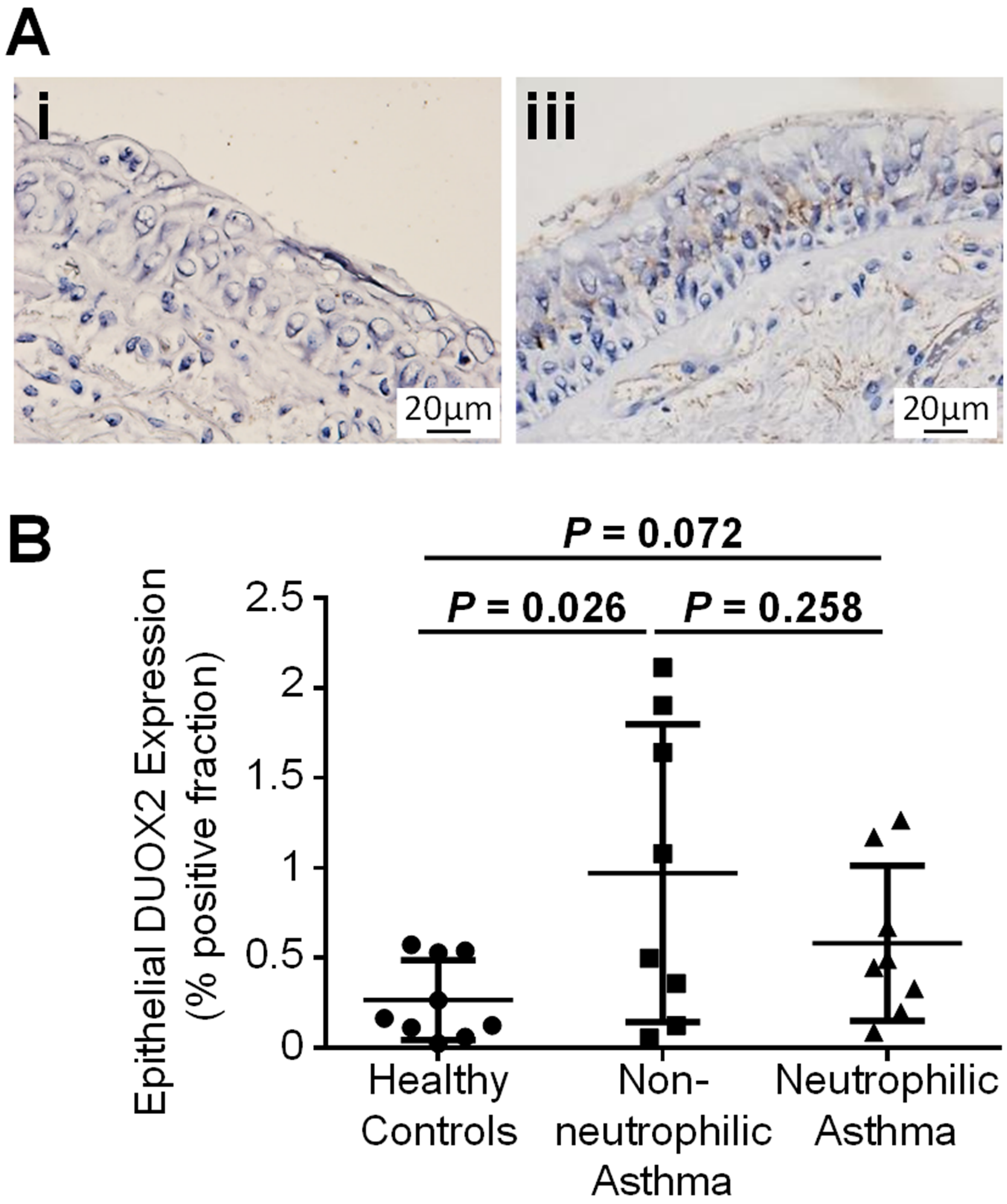

Online supplements are not copyedited prior to posting and the author(s) take full responsibility for the accuracy of all data.
