# Supplementary figures and images for: NADPH Oxidase-4 Overexpression Is Associated With Epithelial Ciliary Dysfunction in Neutrophilic Asthma
Source: Chest. 2016 Feb 2;149(6):1445–59. doi: 10.1016/j.chest.2016.01.024 (PMC4893823; doi:10.1016/j.chest.2016.01.024)

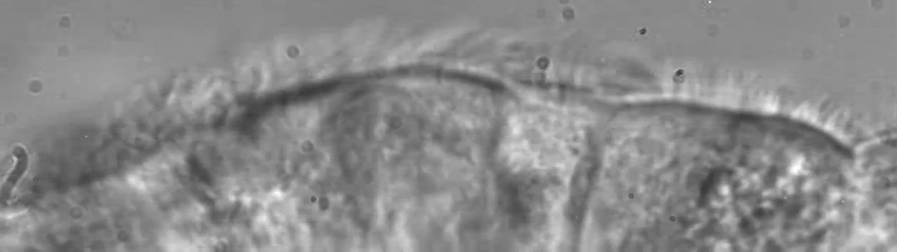

Supplement: Video 1A [file mmc2.jpg]

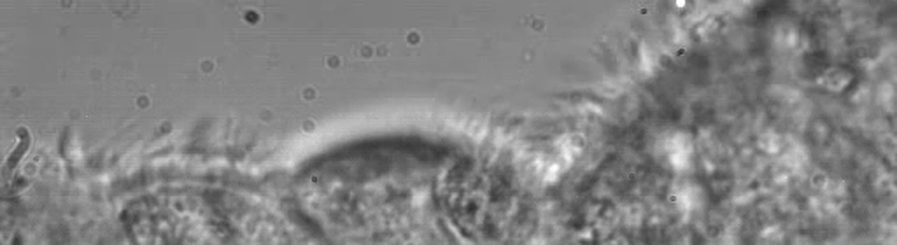

Supplement: Video 1B [file mmc3.jpg]

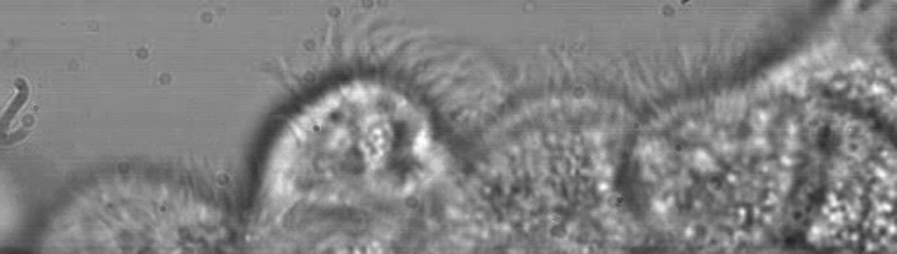

Supplement: Video 1C [file mmc4.jpg]

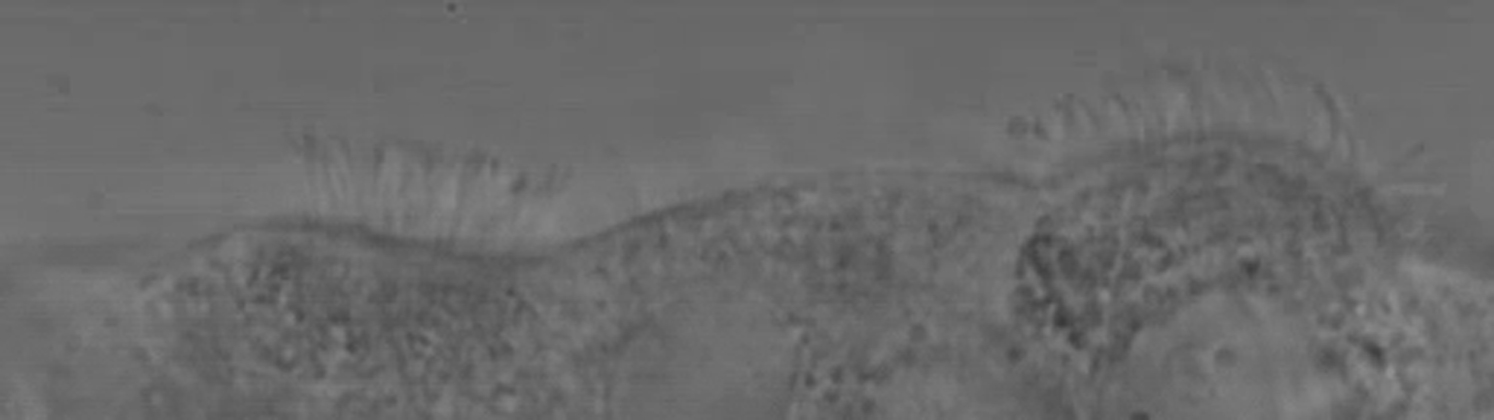

Supplement: Video 1D [file mmc5.jpg]
